# Testing the carbohydrate-insulin model of obesity in a 5-month feeding study: the perils of post-hoc participant exclusions

**DOI:** 10.1038/s41430-020-0658-8

**Published:** 2020-05-20

**Authors:** David S. Ludwig, Kimberly F. Greco, Clement Ma, Cara B. Ebbeling

**Affiliations:** 1 New Balance Foundation Obesity Prevention Center, Boston Children’s Hospital and Harvard Medical School, Boston, MA USA; 2 Institutional Centers for Clinical and Translational Research, Boston Children’s Hospital, Boston, MA USA; 3 Dana-Farber/Boston Children’s Cancer and Blood Disorders Center and Harvard Medical School, Boston, MA USA

**Keywords:** Metabolism, Obesity

## Abstract

A large feeding study reported that total energy expenditure (TEE) was greater on a low- versus high-carbohydrate diet, supporting the carbohydrate-insulin model of obesity. Recently, the validity of this finding was challenged in a *post-hoc* analysis excluding participants with putative non-adherence to the study diets. Here, we show why that analysis, based on a post-randomization variable linked to the outcome, introduced severe confounding bias. With control for confounding, the diet effect on TEE remained strong in a reanalysis. Together with sensitivity analyses demonstrating robustness to plausible levels of non-adherence, these data provide experimental support for a potentially novel metabolic effect of macronutrients that might inform the design of more effective obesity treatment.

## Introduction

Are all calories metabolically alike to the body? According the carbohydrate-insulin model of obesity (CIM) [1, 2], the high insulin-to-glucagon ratio on a high-glycemic load diet shifts substrate partitioning from lean tissue to adipose, lowers the concentration of metabolic fuels in the blood in the late postprandial period, increases hunger and lowers energy expenditure – biological responses that would tend to promote weight gain. Thus, the CIM offers a physiological explanation for why average BMI in many countries increased in the late 20^th^ century as public health guidelines recommended replacement of dietary fat with carbohydrate, and consumption of high-glycemic load foods (chiefly processed grains, potato products and added sugars) increased substantially.

One prediction of the CIM is that, during weight-loss maintenance, total energy expenditure (TEE) would increase with carbohydrate restriction, an effect estimated to be approximately +50 kcal/d for every 10% decrease in dietary carbohydrate as an absolute proportion of total energy intake [2]. Although a recent meta-analysis claimed to find no evidence for this metabolic effect in macronutrient-controlled trials [3], the median duration of included studies was <7 days, an insufficient timeframe to allow for the well-described transient changes that occur over 2 to 3 weeks with reduction in carbohydrate intake [2].

In 2018, our group reported the results of a large feeding study comparing low- (20%), moderate- (40%), and high- (60%) carbohydrate diets throughout 5 months weight-loss maintenance (i.e., with adjustment of dietary energy to prevent weight change). We found that TEE measured by doubly-labeled water (DLW) was approximately 250 kcal/d greater on the low- vs high-carbohydrate diet [4]. If this effect were reproducible and durable, it might translate into substantial weight loss under natural conditions (i.e., without experimental control of dietary intake).

**Fig. 1:**
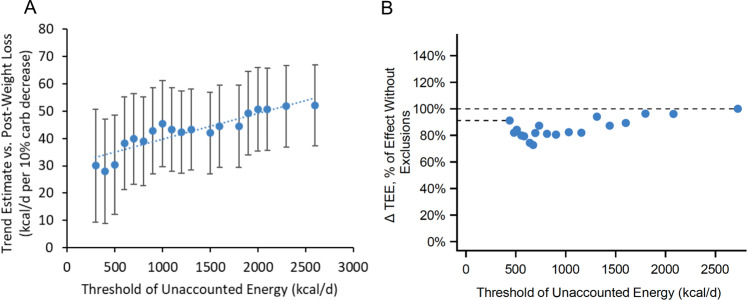
Confounding arising from post-randomization participant exclusion on TEE effect size estimates. TEE data from Ebbeling et al. [4] comparing Low- (20%) vs High- (60%) carbohydrate diets in Intention-to-Treat analyses. **a** Sequential elimination of 50% of participants based on “Unaccounted Energy” (UE, right to left, 1st to 18th of 36 quantiles) suggests a 42% attenuation in effect size. Figure modified from Hall and Guo in accordance with license CC0 https://www.biorxiv.org/content/10.1101/476655v5. The regression statistics were deleted because conditions for regression are not satisfied. (Individual points in this exclusion analysis are not independent of each other. Dashed line should be disregarded for the same reason.) **b** Exclusion analysis performed with final dietary intake and body composition data [7], with adjustment for potential baseline confounders as described in Methods. The results, expressed as a proportion of the diet effect present in the full cohort, indicate a lesser degree of effect attenuation (9%, or approximately one fifth of that observed by Hall and Guo). Qualitatively similar findings (i.e., substantially reduced effect attenuation) were obtained in a Per Protocol analysis (*n* = 104, data not shown).

Soon after publication of this study, one group reanalyzed the publicly available data by eliminating participants according to “Unaccounted Energy” (UE), a calculated variable intended to reflect non-adherence to the test diets [5]. They compared individual levels of energy intake with TEE, adjusting for change in body weight, and sequentially excluded those with high UE as shown in Fig. 1a. In this analysis, the dietary effect on TEE seems to decrease by nearly half, comparing the full cohort represented by the right-most data point with the left-most point from which 50% of participants had been eliminated. This relationship was interpreted as demonstrating that dietary non-adherence invalidated study outcomes due to the dependency of DLW methodology on assumptions about respiratory quotient (RQ).

In a recent report [6], we argued this exclusion analysis, based on post-randomization factors, risks introducing severe bias because of the implicit assumption that UE is causally related to the outcome. In fact, this apparent relationship could be influenced by any baseline or post-randomization factor associated with measurement of both UE and TEE. Once data are excluded in this way, the protection from randomization is lost, and the analyses become subject to the major methodological limitations of observational research, most importantly confounding. Here, we employed conventional epidemiological methods to examine the validity of the reanalysis.

## Methods and results

Statistical analyses were performed using SAS version 9.4 (SAS Institute Inc., Cary, NC). Since individual-level dietary data, a secondary outcome in our original study, were preliminary, we first obtained complete dietary intake [7]. We calculated UE as the absolute value of the difference between energy intake and energy expenditure, correcting for change in body fat mass by isotope dilution (see [7] for details on determination of, and adjustment for, body composition). Using the median value of 438.1 kcal/d, we dichotomized the cohort between those with the highest vs lowest UE, the former comprising those excluded in Panel A.

As shown in Table 1, the excluded group had more males; were younger, heavier and taller; had lost less weight during the weight-loss run-in; and most notably, had higher baseline TEE. They also had a numerically higher baseline insulin-30 (a marker of insulin secretion, an effect modifier) [4]. Each of these covariates would likely be associated with a greater magnitude of TEE outcome in absolute terms, independent of UE.

**Table 1:** Participant characteristics dichotomized according to low- vs high- “Unaccounted Energy” (UE).

Variable	Total (*N* = 145)	Low UE (*N* = 73)	High UE (*N* = 72)	*P* value
Race, *N* (%)				
* White*	114 (78.6%)	59 (80.8%)	55 (76.4%)	0.5537
* Black*	15 (10.3%)	5 (6.8%)	10 (13.9%)	
* Asian*	5 (3.4%)	3 (4.1%)	2 (2.8%)	
* Other*	11 (7.6%)	6 (8.2%)	5 (6.9%)	
Hispanic, *N* (%)	21 (14.5%)	13 (17.8%)	8 (11.1%)	0.2519
Female, *N* (%)	100 (69.0%)	60 (82.2%)	40 (55.6%)	0.0005
Age^a^ Median (IQR)	35.7 (24.1, 51.2)	44.2 (27.0, 52.3)	33.5 (21.7, 49.0)	0.0485^b^
Weight^a^ (kg), Mean (SD)	91.3 (18.3)	87.1 (16.8)	95.6 (18.9)	0.0045
Height^a^ (cm), Mean (SD)	168.0 (10.1)	165.8 (9.6)	170.2 (10.2)	0.0083
BMI^a^ (kg/m^2^), Mean (SD)	32.2 (4.8)	31.5 (4.6)	32.9 (4.9)	0.0965
Body Fat^a^ %, dual-energy x-ray absorptiometry, Mean (SD)	40.7 (6.4)	41.5 (5.8)	39.8 (6.9)	0.0989
Insulin-30^a^ (uIU/ml), Median (IQR)^c^	113.5 (77.2, 169.7)	109.7 (73.9, 155.1)	119.2 (85.8, 173.5)	0.1112^b^
TEE^a^ (kcal/d), Mean (SD)^d^	3008 (718)	2841 (672)	3180 (729)	0.0045
Energy intake at start of Test phase (kcal/d), Mean (SD)	2214 (373)	2189 (343)	2239 (402)	0.4265
Run-in Percent Weight Loss (%), Mean (SD)	10.5 (1.6)	10.9 (1.5)	10.1 (1.6)	0.0007

^a^Pre-weight-loss data were collected prior to the Run-in phase. Mean weight loss during the Run-in was 10.5%. During the subsequent Test diet phase, weight-loss maintenance was achieved by periodic adjustment of dietary energy, as described in [4].

^b^Skewed distribution; *p* value derived from Wilcoxon rank sum test (all other comparisons made with chi-squared and independent t-tests).

^c^Missing data on insulin-30 for two subjects with low UE (total *n* = 143).

^d^Missing data on TEE for three subjects, one with low UE and two with high UE during the Test phase (total *n* = 142).

We repeated the exclusion analysis using repeated measures ANOVA to test the effect of diet on TEE while controlling for the above-mentioned baseline covariates, insulin-30, and study cohort (the latter to control for temporal effects over the 3-year study, consistent with our original analysis plan) [4]. Change in TEE was expressed as kcal/day/kg normalized to the average post-weight loss weight (82 kg). As shown in Fig. 1b, TEE diet effect for the low- vs high-carbohydrate diet declined with sequential exclusions markedly less with covariate adjustment than without adjustment (attenuation of 9% vs 42%). Moreover, there was no difference in TEE diet effect among those in the lowest vs highest category of UE (*p* = 0.75).

## Discussion

By eliminating 50% of the sample with highest UE, only 9% of the originally observed diet effect on TEE might be attributable to non-adherence. Most likely, the amount so attributable would be even less, as the reanalysis is still subject to confounding by post-randomization factors beyond our control. Specifically, individuals who have a bigger than average diet-induced increase in TEE from measurement error (and consequently a larger apparent UE) would be excluded to a greater extent than those with a smaller than average increase from measurement error in the opposite direction. In this fashion, one tail of a distribution curve would be selectively depleted, producing unbalanced (systematic) error with bias toward the null hypothesis. Moreover, the very notion of UE is potentially misleading, because it comprises multiple components of energy balance (intake, expenditure, and body composition) that each have measurement imprecision. An individual might have high UE arising not only from dietary non-adherence, but also cumulative random measurement error. Even in the optimal experimental environment afforded by a metabolic ward, most participants in a 2-week feeding study had UE ≥ 250 kcal/d [8].

The physiological mechanisms relating a low-carbohydrate diet to higher energy expenditure have been considered elsewhere [1, 2, 4, 7] and may involve lower insulin and ghrelin action in adipose tissue, higher glucagon action in non-adipose sites, increased leptin sensitivity in muscle, and multiple hormonal and metabolic signals acting in the brain. Conversely, on a high-carbohydrate diet, thyroid hormone level (though not necessarily hormone sensitivity) and sympathetic nervous system activity are higher. Although more research will be needed to clarify mechanisms, the effects we observed are independent of body composition [7], and likely to involve a major contribution from non-resting energy expenditure, consistent with current understanding of adaptive thermogenesis during weight-loss maintenance [9].

Sensitivity analyses also provide confidence in the validity of the study’s main findings. The higher TEE on the low- vs high-carbohydrate diet remained statistically significant up to 50% non-adherence, using conservative assumptions for RQ [6]. Furthermore, the diet effect on energy requirement was not attenuated when participants with energy intake to expenditure ratio higher *and* lower than average were eliminated (i.e., those at both tails of the distribution) [7]. Finally, new data on energy requirements for weight-loss maintenance show effects commensurate with TEE (about 200 to 300 kcal greater on the low- vs high-carbohydrate diet) [7]. Thus, our study provides support for the CIM and for a potentially novel effect of dietary macronutrients on metabolism. In view of the methodological limitations of all feeding studies, additional research into this macronutrient effect is warranted. More broadly, our current reanalysis highlights the well-described perils of excluding data from an RCT based on post-randomization variables [10].

## References

[1] Ludwig DS (2002). The glycemic index: physiological mechanisms relating to obesity, diabetes, and cardiovascular disease. JAMA..

[2] Ludwig DS, Ebbeling CB (2018). The carbohydrate-insulin model of obesity: beyond “calories in, calories out”. JAMA Int Med.

[3] Hall KD, Guo J (2017). Obesity energetics: body weight regulation and the effects of diet composition. Gastroenterology..

[4] Ebbeling CB, Feldman HA, Klein GL, Wong JMW, Bielak L, Steltz SK (2018). Effects of a low carbohydrate diet on energy expenditure during weight loss maintenance: randomized trial. BMJ..

[5] Hall KD, Guo J, Speakman JR (2019). Do low-carbohydrate diets increase energy expenditure?. Int J Obes (Lond).

[6] Ludwig DS, Lakin PR, Wong WW, Ebbeling CB (2019). Scientific discourse in the era of open science: a response to Hall et al. regarding the Carbohydrate-Insulin Model. Int J Obes (Lond).

[7] Ebbeling CB, Bielak L, Lakin PR, Klein GL, Wong JMW, Luoto PK, et al. Energy requirement is higher during weight-loss maintenance in adults consuming a low- compared with high-carbohydrate diet. J Nutr. 2020, in press. 10.1093/jn/nxaa150 PMC7398766 32470981

[8] Hall KD, Ayuketah A, Brychta R, Cai H, Cassimatis T, Chen KY (2019). Ultra-processed diets cause excess calorie intake and weight gain: an inpatient randomized controlled trial of ad libitum food intake. Cell Metab.

[9] Müller MJ, Enderle J, Bosy-Westphal A (2016). Changes in energy expenditure with weight gain and weight loss in humans. Curr Obes Rep.

[10] Piantadosi S. Random error and bias (Chpt 6) In: Clinical trials: a methodological perspective, 3rd ed. Hoboken, NJ: John Wiley & Sons, Inc.; 2017.

